# Continuing Professional Development ‐ Medical Imaging

**DOI:** 10.1002/jmrs.805

**Published:** 2024-06-17

Maximise your CPD by reading the following selected article and answer the five questions. Please remember to self‐claim your CPD and retain your supporting evidence. Answers will be available via the QR code and published in JMRS – Volume 71, Issue 4, December 2024.

## Medical Imaging – Original Article

### ePortfolios: Enhancing confidence in student radiographers' communication of radiographic anatomy and pathology. A cross‐sectional study

Dolic M , Peng Y , Dhingra K , Lee K , McInerney J . (2024). J Med Radiat Sci 10.1002/jmrs.787 PMC1156940338712980

1. What regulatory body governs the practice of medical radiation practitioners in Australia, setting requirements for radiographers' communication of urgent findings?
  - Australian Medical Association (AMA)
  - Medical Radiation Practice Board of Australia (MRPBA)
  - Royal Australian and New Zealand College of Radiologists (RANZCR)
  - Radiological Society of Australia (RSA)
2. According to the study, what is one of the key responsibilities of radiographers when they identify significant radiographic findings?
  - Write a detailed radiology report
  - Communicate the findings to the patient
  - Convey the findings in a timely manner for patient management
  - Keep the findings to themselves until consulting with a radiologist
3. According to the study, what was identified as the largest contributing factor to the reduction of student confidence when transitioning from academic to clinical environments?
  - Lack of university‐based comprehension of content
  - Pressure associated with the clinical environment
  - Variability in radiographic pathology
  - Poor consolidation of knowledge in clinical settings
4. According to the study, what role do eportfolios play in enhancing students' confidence in identifying and describing radiographic findings?
  - Provide direct feedback from radiologists
  - Simulate real‐world clinical scenarios
  - Encourage long‐term reflection and learning
  - Replace traditional anatomy textbooks
5. What was identified as a limitation of the study?
  - Small sample size
  - Lack of clarity in survey questions
  - Inadequate distribution of eportfolios
  - Absence of qualitative data analysis

## Answers

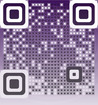

Scan this QR code to find the answers.
